# High-performance n-type flexible inorganic thermoelectric aerogel for energy harvesting

**DOI:** 10.1126/sciadv.ady7679

**Published:** 2026-01-09

**Authors:** Xiaodong Wang, Wenbo Zhu, Yijie Liu, Shuaihang Hou, Li Yin, Jinxuan Cheng, Peng Zhao, Feng Jiang, Sichen Duan, Wenhua Xue, Yumei Wang, Xuesong Leng, Feng Cao, Jun Mao, Mingyu Li, Qian Zhang

**Affiliations:** ^1^ School of Materials Science and Engineering, and Institute of Materials Genome & Big Data, Harbin Institute of Technology, Shenzhen 518055, P.R. China.; ^2^ Institute of Special Environments Physical Sciences, Harbin Institute of Technology, Shenzhen 518055, P.R. China.; ^3^ Sauvage Laboratory for Smart Materials, School of Materials Science and Engineering, Harbin Institute of Technology, Shenzhen 518055, P.R. China.; ^4^ State Key Laboratory of Advanced Welding and Joining, Harbin Institute of Technology, Harbin 150001, P.R. China.; ^5^ School of Science, Harbin Institute of Technology, Shenzhen 518055, P.R. China.; ^6^ Hebei Key Lab of Optic-electronic Information and Materials, The College of Physics Science and Technology, Hebei University, Baoding 071002, P. R. China.; ^7^ Beijing National Laboratory for Condensed Matter Physics, Institute of Physics Chinese Academy of Sciences, Beijing 100190, P.R. China.

## Abstract

Despite their promise as lightweight, ultralow–thermal-conductivity thermoelectric (TE) materials, aerogels have been largely limited to p-type organic or carbon-based systems with modest *zT* < 0.1 at 300 kelvin. Here, we propose a stepwise synthesis strategy that yields the first inorganic aerogel exhibiting state-of-the-art n-type TE performance. Optimized aerogels with 95% porosity exhibit a high power factor of 34.8 microwatts per meter per square kelvin and an ultralow thermal conductivity of 0.061 microwatts per meter per kelvin, resulting in *zT* values of 0.17 at 300 kelvin and 0.24 at 383 kelvin. A vertical TE generator prototype with six TE-aerogel legs achieves a gravimetric output power of 76 microwatts per gram under a Δ*T* of ~60 kelvin. To address brittleness, a polyimide-encapsulated aerogel with bioinspired architecture was developed, achieving a high compressive strength to 1.4 kilopascals while maintaining excellent TE performance. This work establishes a generalizable method for designing high-performance flexible inorganic aerogels, opening more possibilities for lightweight wearable energy harvesting technologies.

## Teaser

A two-step synthesis method was proposed to construct an n-type Ag_2_Se aerogel with record-high thermoelectric performance.

## INTRODUCTION

Wearable thermoelectric generators (WTEGs) hold immense potential to revolutionize healthcare technologies by harvesting body heat and converting it into electricity to power electronics and sensors (*1*, *2*). These devices rely on advanced TE materials with high dimensionless figure of merit *zT* = *S*^2^σ*T*/κ, where σ is the electrical conductivity, *S* the Seebeck coefficient, κ the thermal conductivity, and *T* the absolute temperature. In addition, they must sustain a temperature gradient across the device while maintaining lightweight and comfortable for long-term wearability. Aerogels have emerged as promising candidates in this context due to their interconnected nanoscale networks, offering exceptionally high internal surface areas, ultralow densities, and extremely low thermal conductivity (*3*–*5*). Given these distinctive properties, aerogels are attractive for efficient WTEGs, but high-performance TE aerogels remain scarce (*6*–*13*). Conventional aerogels, such as silica (*14*), carbon (*15*), graphene (*16*), metal oxide (*17*), conductive polymer (*18*, *19*), composite (*20*–*23*), and noble metal aerogels (*24*, *25*), are mostly electrically insulating, leading to an extremely low TE *zT* < 0.1 at room temperature.

In recent years, considerable efforts have been devoted to advancing organic aerogels. However, even widely used poly(3,4-ethylenedioxythiophene):poly(styrenesulfonate) (PEDOT:PSS)-based composites continue to suffer from low *S*, limiting their maximum *zT* to the order of 10^−2^ (*26*–*28*). In contrast, inorganic TE aerogels face distinct challenges, primarily due to suboptimal fabrication techniques and the use of nonconductive surfactants. As a result, the obtained Bi_2–*x*_Sb*_x_*Te_3_ aerogels exhibit low crystallinity, poor phase purity, and low *zT* values, reaching as low as 5 × 10^−3^ at room temperature (*29*). To address these limitations, a stepwise impregnation method was recently proposed to construct a three-dimensional (3D) inorganic TE network with the aid of commercial melamine foam as a template skeleton (*30*). This approach markedly enhanced the room-temperature *zT* value to ~0.11, effectively narrowing the performance gap between 3D network materials and commercial bulk counterparts by nearly an order of magnitude.

Here, we propose a step-by-step synthesis strategy to construct a robust, well-connected 3D Ag_2_Se aerogel network without relying on organic templates, which are typically nonconductive. The obtained highly porous Ag_2_Se aerogel with an exceptional porosity of 95 to 99% exhibits an extremely low density of 0.04 to 0.54 g cm^−3^ and an ultralow thermal conductivity of 0.027 to 0.071 W m^−1^ K^−1^, contributing to an enhanced *zT* value of 0.17 at room temperature and establishing a sufficient temperature gradient between the hot and cold ends. In addition, polyimide (PI) encapsulation transforms the brittle Ag_2_Se network into a mechanically robust and tunable aerogel composite. A vertical TE device comprising six Ag_2_Se aerogel legs was assembled, achieving a high output power-to-weight ratio of 76 μW g^−1^ at a hot-side temperature of 353 K.

## RESULTS

**Fig. 1.**
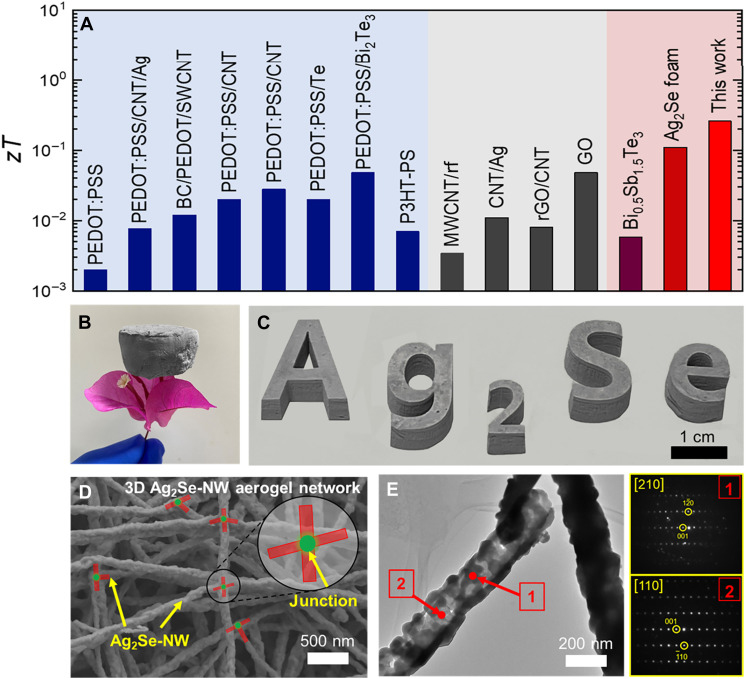
Performance comparable and morphology characterization of Ag_2_Se-NW aerogel. (**A**) Comparison of the *zT* values of the optimized Ag_2_Se aerogel and other reported TE aerogels (*13*, *19*–*21*, *26*, *29*, *30*, *51*, *58*–*62*). SWCNT, single-walled carbon nanotube. rGO, reduced graphene oxide. Photos of (**B**) light Ag_2_Se aerogel and (**C**) Ag_2_Se aerogel with different shapes. (**D**) Scanning electron microscopy (SEM) and (**E**) transmission electron microscope (TEM) images of Ag_2_Se-aerogel-95 together with selected-area electron diffraction. P3HT-PS, poly(3-hexylthiophene-2,5-diyl)-b-poly(styrene).

The Ag_2_Se aerogel exhibits both high electrical conductivity and low thermal conductivity, achieving a state-of-the-art room-temperature *zT* value of 0.17. This performance surpasses that of all previously reported organic-based and carbon nanotube (CNT)–based aerogel composites, as well as Bi_0.5_Sb_1.5_Te_3_ aerogels (see Fig. 1A). The key advantages of ultralow density and facile processability are respectively presented in Fig. 1 (B and C). The microstructure of the Ag_2_Se aerogel is shown in Fig. 1 (D and E). A well-interconnected 3D porous architecture is observed, which is composed of uniformly distributed nanowires (NWs) joined through chemically bonded junctions. After the selenization reaction and supercritical drying, the Ag_2_Se aerogel preserves the architecture of the original Ag aerogel network (see fig. S1), without any observable structural collapse or degradation. Furthermore, transmission electron microscope (TEM) analysis verifies the NW morphology of Ag_2_Se with an average diameter of ~229 nm (Fig. 1E). These NWs consist of randomly oriented columnar grains aligned perpendicular to their longitudinal axis, consistent with previous reports (*29*). This structural feature is consistent with previous findings (*31*). The distinct and periodic electron diffraction patterns in Fig. 1E validate the crystallization of Ag_2_Se (PDF#24-1041).

**Fig. 2.**
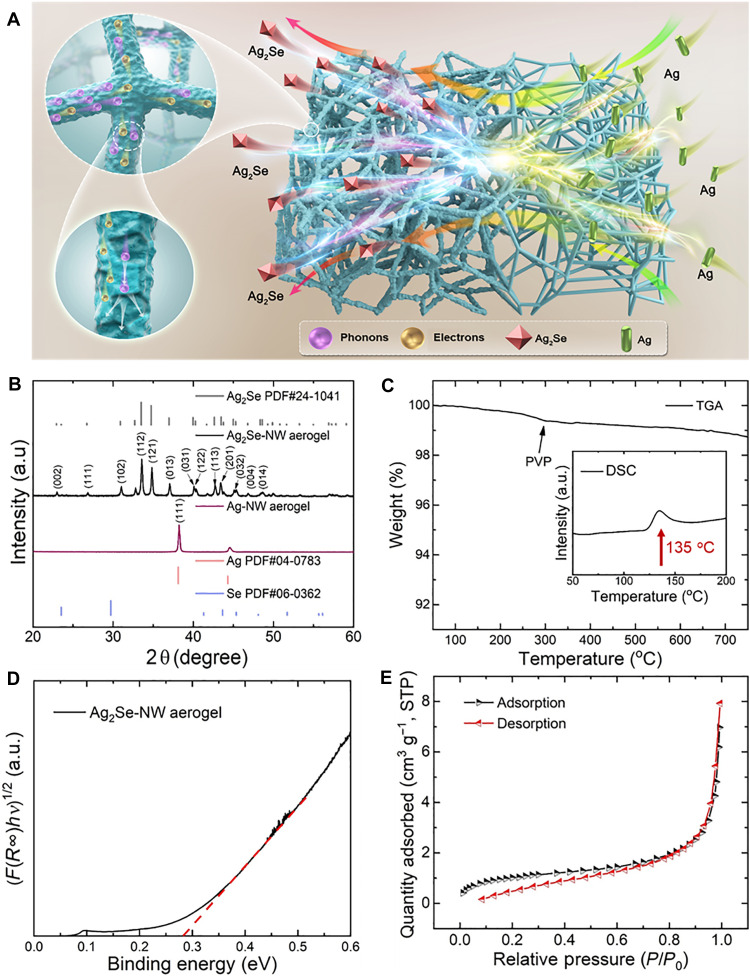
Synthesis and chemical structural characterization of Ag_2_Se-NW aerogel. (**A**) Schematic of the synthetic route of Ag_2_Se aerogel. (**B**) X-ray diffraction (XRD) patterns of the Ag-NW aerogel and the Ag_2_Se aerogel. a.u., arbitrary units. (**C**) Thermogravimetric analysis (TGA) curve of the Ag_2_Se aerogel from 50° to 750°C with the differential scanning calorimetry (DSC) curve inserted. (**D**) (*F*(*R*)*h*ν)^1/2^ versus photon energy (*h*ν), where *F* is the Kubelka-Munk function of the diffuse reflectance *R*. (**E**) N_2_ adsorption/desorption isotherms for the Ag_2_Se aerogel at 77 K. The specific surface area is 4.4 m^2^ g^−1^ calculated by Brunauer-Emmett-Teller (BET) method. STP, standard temperature and pressure.

To further investigate the high-performance Ag_2_Se aerogel, we schematically illustrated the synthetic pathway in Fig. 2A. The Ag_2_Se aerogel was synthesized by a surface selenization reaction of a preformed Ag aerogel (*32*). Initially, a 3D cross-linked Ag aerogel network was synthesized by the hydrothermal method (*24*), exhibiting a high porosity of 95 to 99.5% and a low density of 0.04 to 0.54 g cm^−3^. The Ag aerogel was then converted into the Ag_2_Se aerogel by a selenization reaction at room temperature, where Se powder reacted in Na_2_S solution to generate (SSe_2_)^2−^ or (SSe*_x_*)^2−^ species, which facilitated the efficient conversion of elemental Ag into Ag_2_Se within 60 s. Last, CO_2_ supercritical drying was used to prevent volumetric shrinkage caused by solvent surface tension and preserve the integrity of the 3D hydrogel network (*31*). The intact 3D structure ensures the efficient electron transport, while the high density of pores and grain boundaries inhibit the propagation of phonons.

The representative x-ray diffraction (XRD) patterns of Ag and Ag_2_Se aerogels are presented in Fig. 2B. The diffraction peaks can be well indexed to the standard peaks of Ag (PDF#04-0783) and Ag_2_Se (PDF#24–1041) (*33*–*35*). No diffraction peaks of elemental Ag or Ag_2_S were observed in the Ag_2_Se aerogel within the detection range, indicating nearly complete conversion from Ag to Ag_2_Se. While aerogels are generally considered to have low crystallinity due to their high surface area and low-density structures, the carefully controlled selenization process in this work enables the formation of highly crystalline Ag_2_Se NWs. To evaluate the thermal stability of the Ag_2_Se aerogel, we performed thermogravimetric analysis (TGA). As illustrated in Fig. 2C, it exhibits good thermal stability with a weight loss of less than 1.5 wt % upon heating to 750°C. The phase-transition behavior was further characterized by differential scanning calorimetry (DSC). As shown in the inset of Fig. 2C, a phase transition from orthogonal to cubic phase occurs at the temperature of 135°C, which is consistent with the bulk material (*36*). The optical bandgap of Ag_2_Se aerogel is ~0.27 eV, as provided in Fig. 2D, which is slightly larger than that of its bulk counterpart (*37*). Nitrogen adsorption-desorption analysis (Fig. 2E) reveals a typical type-I adsorption isotherm, with a Brunauer-Emmett-Teller (BET) specific surface area of 4.4 m^2^ g^−1^, which is slightly lower than that of Ag aerogel (see fig. S2) and much lower than that of cellulose aerogel (*38*, *39*), SiC aerogel (*40*), graphene aerogel, (*41*), and CNT aerogel (*42*). Table S1 presents the comparison of the specific surface area measured by BET and mercury intrusion porosimetry method.

**Fig. 3.**
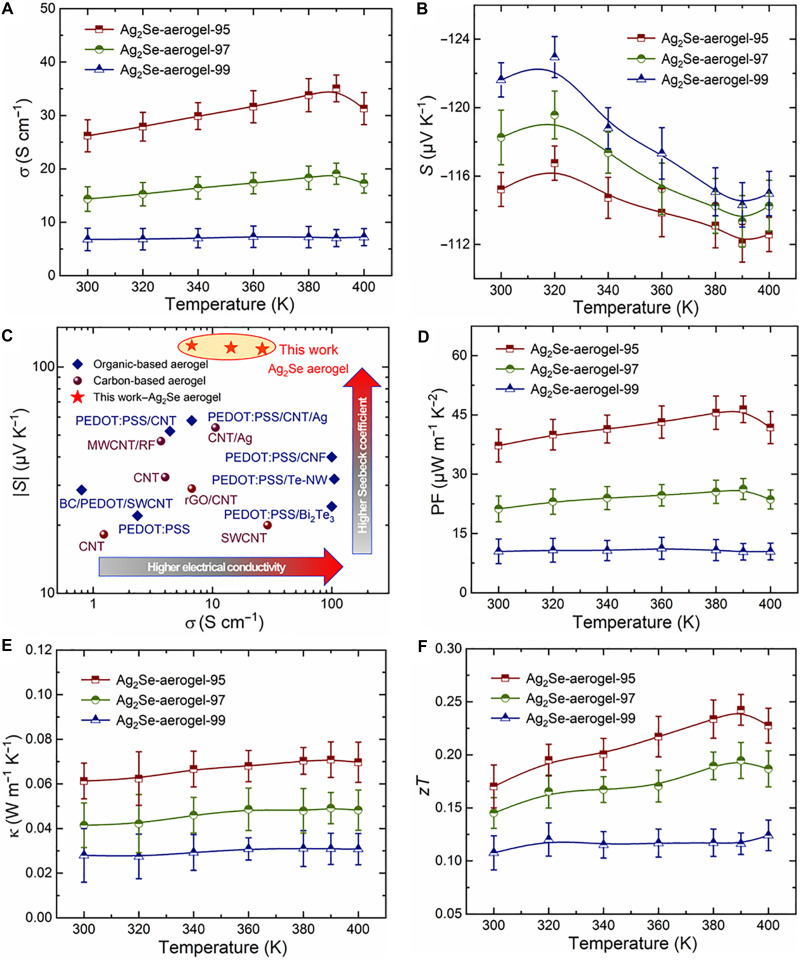
Temperature-dependent TE properties of Ag_2_Se-NW aerogel. Temperature-dependent (**A**) electrical conductivity (σ) and (**B**) Seebeck coefficient (*S*) of the Ag_2_Se aerogels with different porosity. (**C**) The absolute value of Seebeck coefficient (|*S*|) versus room-temperature electrical conductivity (σ) for Ag_2_Se aerogel compared with that of previously reported organic- and CNT-based aerogel TE materials. BC, bacterial cellulose. Temperature-dependent (**D**) power factor (*PF*), (**E**) thermal conductivity (κ), and (**F**) figure-of-merit *zT* of Ag_2_Se aerogels with different porosity.

Figure 3A presents the temperature-dependent electrical conductivity of Ag_2_Se aerogels with varying porosities, denoted as Ag_2_Se-aerogel-X, where X = 95, 97, and 99 corresponds to the porosity (%). The electrical conductivity increased from 6.8 S cm^−1^ for Ag_2_Se-aerogel-99 to 26.2 S cm^−1^ for Ag_2_Se-aerogel-95. With increasing temperature, the electrical conductivity increased, probably due to the thermally activated carrier concentration, reaching a maximum value of 35.1 S cm^−1^ at 390 K for Ag_2_Se-aerogel-95. For samples with different porosity, the Seebeck coefficient fluctuates between values of −112 and −122 μV K^−1^ below 390 K, as shown in Fig. 3B, which is slightly lower than that of the bulk Ag_2_Se (−140 μV K^−1^) (*43*, *44*). It can be arose from Ag enrichment at NW junctions. Scanning electron microscopy–energy dispersive spectrometer (SEM–EDS) mapping (fig. S3) confirms localized Ag-rich regions at junction points, which increases the carrier concentration and suppresses *S*. A noticeable change beyond 390 K may be associated with the known phase transition in Ag_2_Se (*45*). Despite this, the combination of a chemically cross-linked 3D network and well-maintained crystalline phases endows the Ag_2_Se aerogel with electrical conductivity and Seebeck coefficient compared to other organic- and CNT-based TE aerogels, as depicted in Fig. 3C. Combining the results of the electrical conductivity and the Seebeck coefficient, the temperature-dependent power factor is depicted in Fig. 3D. Lower porosity leads to a higher power factor due to the increased electrical conductivity. The maximum power factor is 34.8 μW m^–1^K^–2^ at 390 K for Ag_2_Se-aerogel-95.

The unique aerogel structure also imparts excellent thermal insulation performance. Figure 3E displays the temperature-dependent thermal conductivity of Ag_2_Se aerogel. The room-temperature thermal conductivity of Ag_2_Se-aerogel-99 is as low as 0.027 W m^−1^ K^−1^, approaching that of air (0.026 W m^−1^ K^−1^). With decreasing porosity and increasing density (from 0.04 to 0.54 g cm^−3^), the thermal conductivity gradually increases, ranging from 0.027 to 0.071 W m^−1^ K^−1^ over 300 to 400 K. The obtained thermal conductivities are comparable to those of state-of-the-art thermally insulating aerogels, including polyaniline (PANi)/CNT aerogel (0.023 W m^−1^ K^−1^) (*21*), PEDOT:PSS/CNT aerogel (0.093 W m^−1^ K^−1^) (*21*), PEDOT:PSS/Bi_2_Te_3_ NW (0.047 W m^−1^ K^−1^) (*46*), and others (*13*, *47*). Table S2 provides a comprehensive comparison of method, density, electrical conductivity (σ), Seebeck coefficient (*S*), thermal conductivity (κ), and *zT* value for the Ag_2_Se-NW aerogel and other reported aerogel materials. This decoupled optimization of electrical and thermal transport yields unprecedented *zT* values for inorganic aerogels. Specifically, the Ag_2_Se-aerogel-95 sample achieves a *zT* of 0.17 at room temperature, reaching a peak value of 0.24 at 383 K (see Fig. 3F). Although this *zT* value is still much lower than its bulk counterpart (*zT* ~ 1) (*45*), it represents a benchmark for inorganic aerogels. The successful synthesis and thermal stability of this optimized aerogel further validate its potential for practical TE applications.

**Fig. 4.**
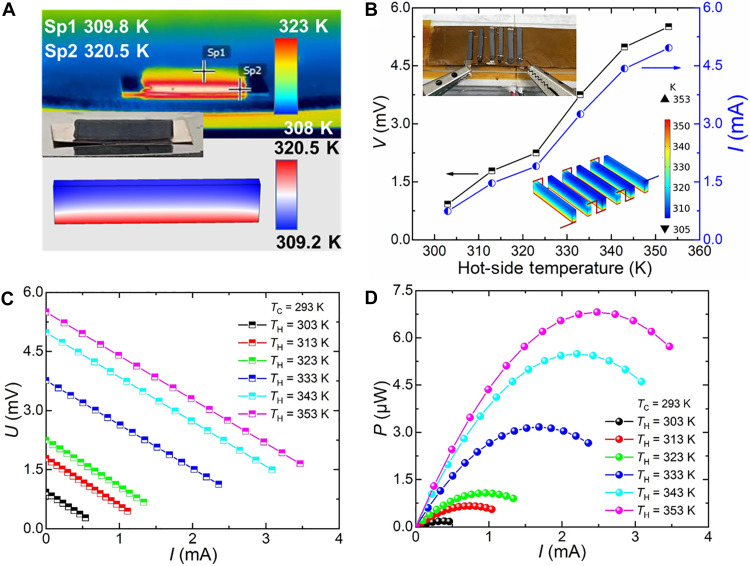
Output performance of Ag_2_Se-based aerogel TE generator. (**A**) The infrared image (up) and theoretical simulation image (down) of a single TE leg (15 mm by 3 mm by 1 mm) on a hot plate maintained at a temperature of 323 K. The optical photograph of the TE leg is inserted. (**B**) Hot-side temperature-dependent open-circuit voltage (*V*) (left) and short-circuit current (*I*) (right) for a six-leg TE generator. The temperature distribution of the aerogel-based generator at the hot-side temperature of 353 K is inserted. Current-dependent (**C**) output voltage (*U*) and (**D**) output power (*P*) with different hot-side temperatures.

The Ag_2_Se aerogel demonstrates outstanding temperature gradient retention capability. The vertical temperature distribution across the aerogel was visualized using an infrared thermal imaging, as shown in Fig. 4A with the corresponding optical image provided in the insert. When subjected to a hot-side temperature of 323 K and ambient temperature of 298 K, the Ag_2_Se aerogel maintained a temperature difference of 10.7 K across its structure. To quantitatively address the duration of temperature gradient maintenance across the Ag_2_Se aerogel with the size of 15 mm by 3 mm by 1 mm, we conducted long-term stability tests under steady-state conditions. The results demonstrate that the temperature difference with ~10.7 K can maintain for more than 10 hours (figs. S5 and S6), confirming our aerogel’s capability to establish and preserve large, stable thermal gradients essential for TE applications. The temperature gradient utilization ratio (φ_thermal_) for an individual Ag_2_Se aerogel leg is notably high, reaching ∼42.8% (see table S3), based on the following equation: φ_thermal_ = Δ*T*/Δ*T*_theory_, where Δ*T* is the practical temperature difference across the aerogel leg and Δ*T*_theory_ is the temperature difference between the heat source and the ambient (*48*). In contrast, conventional bulk TE generators with cooling fins exhibit a much lower temperature gradient Δ*T* ≈ 1 K, which corresponds to a φ_thermal_ ≈ 10% (*49*). All these results prove our aerogel’s capability to establish and preserve large, stable thermal gradients essential for TE applications. It is critical to increase Δ*T* through structure design for a high output performance (*50*). This high φ_thermal_ realized in this work was primarily attributed to the low thermal conductivity of Ag_2_Se aerogel. The experimentally measured Δ*T* aligns closely with that of the theoretical simulation results (see Fig. 4A), confirming the aerogel’s potential for harvesting electricity from vertical temperature gradients.

Leveraging this thermal efficiency, we constructed a vertical thermoelectric generator (TEG) composed of six Ag_2_Se aerogel legs connected in series using copper wires and silver paste (see insert of Fig. 4B). To investigate the impact of electrode geometry on device performance, we conducted finite element simulation using copper wires of varying widths (0.1, 1, and 15 mm). The simulation results (tables S4 and S5 and fig. S7) reveal that increased electrode width leads to a reduction in output voltage, primarily due to the diminished temperature gradient across the aerogel legs caused by the enhanced thermal conduction of the broader electrodes. Experimentally, the open-circuit voltage and short-circuit current of the TEG as a function of the hot-side temperature (*T*_H_) are shown in Fig. 4B. The open-circuit voltage increases rapidly with rising *T*_H_, reaching a peak of ~5.5 mV at *T*_H_ = 353 K, attributed to the enlarged effective temperature difference across the aerogel legs. The output performances (*I*-*V* and *I*-*P* curves) of the TEG under different *T*_H_ were tested and shown in Fig. 4 (C and D). The maximum output power improves with increasing *T*_H_, achieving a maximum of ~6.8 μW at *T*_H_ = 353 K. The output power per weight of the TEG also exhibits a high value of ~76 μW g^−1^ at *T*_H_ = 353 K among all the aerogel-based TE devices (*21*, *23*, *26*, *34*, *51*, *52*). This result highlights the exceptional suitability of porous Ag_2_Se aerogels for lightweight and efficient TE energy conversion.

**Fig. 5.**
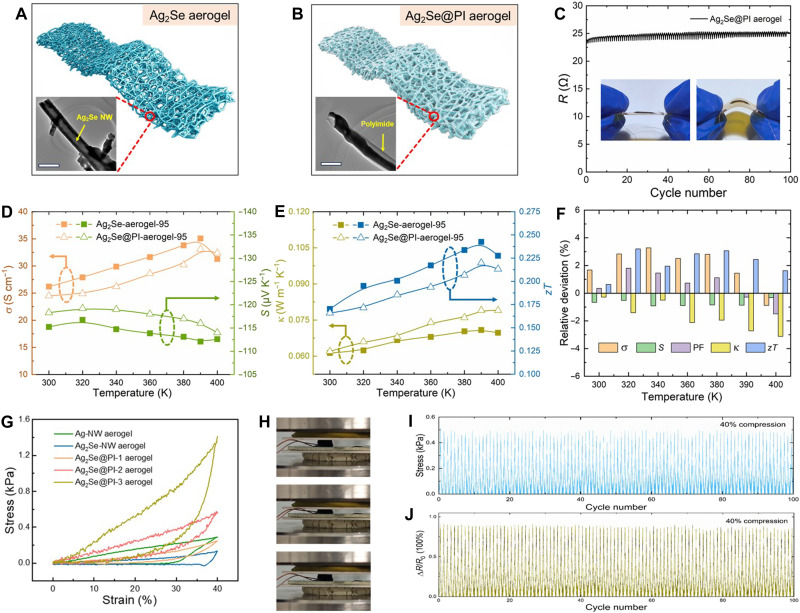
Mechanical and TE characterization of Ag_2_Se-based aerogel materials. Schematic structure and corresponding TEM of (**A**) Ag_2_Se aerogel and (**B**) Ag_2_Se@PI aerogel. (**C**) The resistance (*R*) of Ag_2_Se@PI aerogel versus bending cycles at a bending radius of 5 mm. (**D**) The electrical conductivity (σ; left) and Seebeck coefficient (*S*; right) and (**E**) thermal conductivity (κ; left) and figure-of-merit *zT* (right) of the Ag_2_Se-aerogel-95 and Ag_2_Se@PI-aerogel-95. (**F**) The relative deviation of the TE parameter between Ag_2_Se-aerogel-95 and Ag_2_Se@PI-aerogel-95. (**G**) Stress-strain curves of Ag_2_Se aerogels encapsulated with different PI contents (Ag_2_Se@PI-1, Ag_2_Se@PI-2, and Ag_2_Se@PI-3) under 40% compressive strain. Uncoated Ag-NW and Ag_2_Se-NW aerogels are shown for comparison. (**H**) The photos of cyclic compression tests. The (**I**) stress and (**J**) Δ*R*/*R*_0_ of Ag_2_Se@PI aerogel versus bending cycles.

To further enhance the mechanical durability, we fabricated a PI-encapsulated Ag_2_Se aerogel through a bioinspired hierarchical architecture. High-resolution TEM images (insets of Fig. 5, A and B) confirm the uniform encapsulation of the Ag_2_Se-NW network by PI polymer. This conformal coating imparts polymer-derived flexibility to the aerogel while preserving its high TE properties, forming a robust yet deformable network, which is ascribed to PI’s exceptional tensile strength exceeding 400 MPa and its anisotropic thermal expansion coefficients. The synergistic combination of covalent bonding–dominated interfacial adhesion and tailored elastic modulus could facilitate efficient stress redistribution within the composite matrix, particularly under extreme conditions involving thermal cycling from −269° to 500°C (*53*). First, to evaluate mechanical durability under flexural deformation, we monitored the electrical resistance evolution of the Ag_2_Se@PI aerogel with the size of 15 mm by 3 mm by 1 mm under a bending radius of 5 mm (Fig. 5C). The Ag_2_Se@PI aerogel exhibited negligible resistance fluctuation (±2.1%) over 100 cycles, attributed to its porous framework redistributing stress and the compliant PI coating.

The comparison of the key TE parameters, including electrical conductivity (σ), Seebeck coefficient (*S*), power factor (*PF*), thermal conductivity (κ), and figure of merit (*zT*), between the pristine Ag_2_Se-aerogel-95 and the PI-coated Ag_2_Se@PI-aerogel-95 has been investigated, and the result was shown in Fig. 5 (D to F) and fig. S9. We can see that the PI coating slightly reduces the electrical conductivity (σ) and increases the Seebeck coefficient (*S*) while leaving the thermal conductivity (κ) nearly unchanged. As a result, the overall change in *zT* is negligible. The minimal reduction in electrical conductivity (σ) is due to our encapsulation strategy by controlling the concentration of PI. The PI forms a thin (about 10 to 20 nm), conformal skin on the macroscopic aerogel monolith (see TEM image in Fig. 5B) rather than penetrating deeply and coating every individual NW junction. This preserves the percolating conductive network of the Ag_2_Se NWs while providing crucial mechanical reinforcement at the macroscale. In addition, the maximum relative deviation across all measured TE parameters is just 3.6%, demonstrating that the PI coating enhances mechanical robustness without compromising the TE performance of the Ag_2_Se aerogel.

Furthermore, to evaluate the effect of PI content on mechanical properties, we conducted monotonic compression tests up to 40% strain on Ag_2_Se aerogels encapsulated with different PI contents (Ag_2_Se@PI-1, Ag_2_Se@PI-2, and Ag_2_Se@PI-3). Uncoated Ag-NW and Ag_2_Se-NW aerogels were shown for comparison, and the results were shown in Fig. 5D. In addition, the corresponding deformation images were shown in Fig. 5H. Pristine Ag-NW aerogels showed a compressive modulus of ~0.30 kPa. After selenization, the modulus dropped to ~0.14 kPa due to brittleness. PI coating restored the modulus to ~0.30 kPa and improved recoverability. Increasing PI content enhanced compressive strength over 10-fold, from 0.14 kPa (uncoated Ag_2_Se) to 1.4 kPa at 40% strain. The aerogel demonstrated near-complete shape recovery after unloading, with a tissue-mimetic compressive modulus of ~1.4 kPa, enabling conformal integration with irregular surfaces (*54*). We also conducted microstructure analysis. SEM images (fig. S8) show that the PI layer helps prevent crack propagation, allowing reversible deformation. This makes the aerogel stronger, more resilient, and more robust than both the original Ag-NW and uncoated Ag_2_Se-NW aerogels.

The movie for cycle testing was also provided in the Supplementary Materials. Cyclic compression tests under 40% strain (Fig. 5I) demonstrated mechanical resilience with 96.5% stress retention, consistent with that shown in Fig. 5G. This performance surpasses conventional brittle aerogels, as the PI coating suppresses crack propagation through interfacial energy dissipation (*55*). The corresponding resistance (see Fig. 5J) exhibited an initial 12% attenuation at cycle onset and then maintained exceptional stability with <3% variation throughout 100 cycles, resulting from the strain-induced microstructural homogenization and effective crack suppression by the PI coating. The aerogel’s near-complete shape recovery and ultralow hysteresis align with flexible electronic requirements for conformal integration on curvilinear surfaces. PI encapsulation transforms the brittle Ag_2_Se network into a mechanically robust and tunable aerogel composite. These metrics collectively validate the aerogel’s suitability for flexible electronics operating under dynamic mechanical and thermal loads (*56*).

By adjusting the amount of polyvinyl pyrrolidone (PVP) surfactant during sol-gel synthesis, we tuned the NW diameter from 201 ± 20 to 312 ± 28 nm for Ag NWs, which expanded to 225 ± 21 to 396 ± 44 nm after selenization (figs. S10 and S11). In addition, the influence of fiber diameter on the mechanical properties of aerogels was also explored, and the relevant results and detailed discussions were shown in the figs. S12 and S13. The maximum compressive strengths of 2.06 kPa (60% strain) of 396-nm-diameter Ag_2_Se@PI aerogel were achieved. Our results (fig. S14) show that thinner NWs increase phonon scattering, which can lower thermal conductivity but may also reduce electrical conductivity due to more electron scattering. Optimizing the NW diameter is thus a promising way to improve the aerogel’s TE performance.

Last, the dual strategies, diameter amplification and PI confinement, synergistically transform brittle Ag_2_Se into a mechanically adaptive ultralight material. The tunable strength (0.09 to 2.06 kPa) coupled with fatigue resistance directly addresses the core mechanical challenges in wearable TEs, establishing a robust framework for next-generation flexible energy harvesters.

## DISCUSSION

In summary, we developed the first n-type inorganic aerogel TE material based on Ag_2_Se-NW networks. Through a stepwise synthesis approach, integrating 3D chemical crosslinking of silver NWs, rapid selenization, and CO_2_ supercritical drying, we achieved highly pure, structurally continuous Ag_2_Se networks with programmable porosity. The synergistic preservation of interconnected charge transport pathways and intense phonon scattering in the 99% porosity aerogel simultaneously enhance electrical conductivity to 1.8 × 10^3^ S m^−1^ while suppressing thermal conductivity to 0.027 W m^−1^ K^−1^ at 300 K. This dual optimization yields an impressive room-temperature *zT* of 0.17, escalating to 0.24 at 383 K, which is the highest value among all reported TE aerogels. Furthermore, PI-encapsulated (Ag_2_Se@PI) aerogels maintain mechanical stability under cyclic loading without sacrificing the TE performance, and prototype devices achieve a gravimetric power density of 76 μW g^−1^ at a temperature gradient of 60 K. This work introduces a straightforward yet effective strategy for fabricating high-performance inorganic TE aerogels, opening more possibilities for self-powered wearable electronics.

This advanced methodology for fabricating high-performance n-type TE aerogels could accelerate the development of efficient wearable energy harvesters and ultrasensitive sensor systems. The material’s programmable porosity, robust mechanical stability under cyclic stress, and uncompromised TE performance offer high potential for next-generation wearable electronics, soft robotics, and artificial skin. Furthermore, the readily scalable two-step synthesis method establishes a versatile platform for engineering high-efficiency inorganic TE architectures beyond conventional bulk materials, opening avenues for lightweight, self-sustaining microdevices.

## MATERIALS AND METHODS

### Synthesis Ag NW aerogel

Ag-NW aerogels were synthesized according to a method reported in the literature (*57*). For a typical synthesis, 48 ml of ethylene glycol containing AgNO_3_ (60 mM) and PVP (*M*_W_ = 55000, 0.03 mM) was added into a polytetrafluoroethylene reactor and preheated at 50°C to initiate the crystallization of AgCl seeds. Subsequently, 4.8 ml of iron(III) chloride (FeCl_3_; 4 mM) and fiber-based surfactants were added to the solution and stirred for 5 min. Crucially, the pH is maintained at 10.5 using NH_3_⋅H_2_O throughout to stabilize NW surfaces and prevent aggregation. The mixture was then transferred into a reaction kettle and heated at 120°C for 30 min, followed by further heating at 150°C for 2 hours to form a wet gel of highly cross-linked Ag NWs. The Ag NW was immersed in the deionized water with a flowing speed of 10 ml⋅min^−1^. After washing with deionized water, the cleaned Ag-NW alcogel was performed solvent replacement by ethanol. Last, the Ag-NW alcogel was dried using CO_2_ supercritical fluid drying at 35°C and 8 MPa for 1 hour, resulting in the formation of a lightweight Ag-NW aerogel. By controlling the amount of PVP added during the reaction process (0.1, 0.12, 0.13, 0.16, and 0.19 g), the diameter of synthesized Ag NWs can be effectively controlled, thereby controlling the diameter of Ag_2_Se NWs.

### Synthesis Ag_2_Se-NW aerogel

Ag_2_Se aerogel was fabricated by selenization reaction to convert Ag into Ag_2_Se according to the previously reported literature (*29*). The selenization was carried out at 298 K for 30 min, with a Na_2_S-to-Se molar ratio of 3.2:1. The CO_2_ drying process was conducted under a pressure of 8 MPa. The Ag_2_Se@PI aerogel is obtained by adding a certain amount of PI polymer to the reaction solution forming a 5, 10, and 15 wt % PI solutions, named Ag_2_Se@PI-1, Ag_2_Se@PI-2, and Ag_2_Se@PI-3 aerogels, allowing it to rest for 30 min, followed by a CO_2_ supercritical drying process. The Ag-NW and Ag_2_Se-NW aerogels could be obtained by cutting into the desired shapes and sizes using an ultraviolet laser. Different porosity (ϕ) of Ag_2_Se aerogel was obtained by increasing the AgNO_3_ raw content and cold press strategy and calculated by the following formula: ϕ = (1 –ρ/ρ_T_) × 100%, where ρ is the measured density and ρ_T_ is the theoretical density of bulk Ag_2_Se.The Materials and Methods should provide sufficient information to allow replication of the results. Begin with a section titled Experimental Design describing the objectives and design of the study and prespecified components.

### Characterization

The SEM image was observed on the MIRA-LMH II (TESCAN) equipped with an EDS detector. The XRD pattern was examined by Rigaku (MiniFlex-600) with Cu Kα radiation. The TEM images were acquired at 200 kV using a JEM-2100F JEOL. The TGA and DSC were both characterized on a NETZSCH STA 2500 Regulus in a N_2_ atmosphere with the sample heated in an Al_2_O_3_ crucible at a heating rate of 10°C min^−1^. Optical bandgap was obtained on a Fourier transform infrared (IR) reflectance spectrometer (Nicolet iS50, Thermo Fisher Scientific) equipped with an integrating sphere coated with gold (Pike). The BET surface area was determined using a Micromeritics 3Flex analyzer (Micromeritics ASAP 2460). The surface radiative temperature distribution of the Ag_2_Se aerogel network was characterized via a thermal imaging camera (FLIR T460). The cyclic electrical and stress tests were conducted in situ through an electronic-mechanical testing machine (AGX-V, Shimadzu, Japan) equipped with a 100-N sensor having an accuracy of ±0.3%.

### TE property measurements

The electrical conductivity (σ) and Seebeck coefficient (*S*) were measured on a commercial ZEM-3 setup (ULVAC-RIKO Inc., Japan) from room temperature to 403 K. The thermal conductivity (κ) is calculated by κ = ρ·*D*·*C*_p_, where *D* is the thermal diffusivity measured by the laser flash apparatus (LFA-457, NETZSCH, Germany), ρ is measured on the basis of the Archimedes method (see Supplementary Materials), and *C*_p_ is the heat capacity and measured to be 0.254 J g^−1^ K^−1^ (see fig. S4) based on a DSC (DSC-404F3, NETZSCH), which is very close to the calculated value of 0.253 J g^−1^ K^−1^.

### Assembly and measurement of the TE generator

The TE generator has been fabricated by connecting the Ag_2_Se aerogel network using copper wire and silver paste in series. The dimension of the aerogel legs is about 1 mm by 3 mm by 15 mm. The output performance of the TE generator at the temperature gradient (Δ*T*) from 0 to 60 K was measured by a home-made apparatus connected with two electricity meters (2400 and 2182, Keithley).

## Supplementary Materials

### The PDF file includes:

Figs. S1 to S14 Tables S1 to S5 Supplementary Text Legend for movie S1 References
