# Supplementary material for: High-performance n-type flexible inorganic thermoelectric aerogel for energy harvesting
Source: Sci Adv. 2026 Jan 9;12(2):eady7679. doi: 10.1126/sciadv.ady7679 (PMC12787517; doi:10.1126/sciadv.ady7679)
Supplement: Supplementary file 1 — Figs. S1 to S14 Tables S1 to S5 Supplementary Text Legend for movie S1 References [file sciadv.ady7679_sm.pdf]

Science Advances
AAAS

Supplementary Materials for

High-performance n-type flexible inorganic thermoelectric aerogel for energy harvesting

Xiaodong Wang *et al.*

Corresponding author: Feng Cao, caofeng@hit.edu.cn; Jun Mao, maojun@hit.edu.cn; Mingyu Li, myli@hit.edu.cn; Qian Zhang, zhangqf@hit.edu.cn

Sci. Adv. **12**, eady7679 (2026)
DOI: 10.1126/sciadv.ady7679

The PDF file includes:

Figs. S1 to S14
Tables S1 to S5
Supplementary Text
Legend for movie S1
References

Other Supplementary Material for this manuscript includes the following:

Movie S1

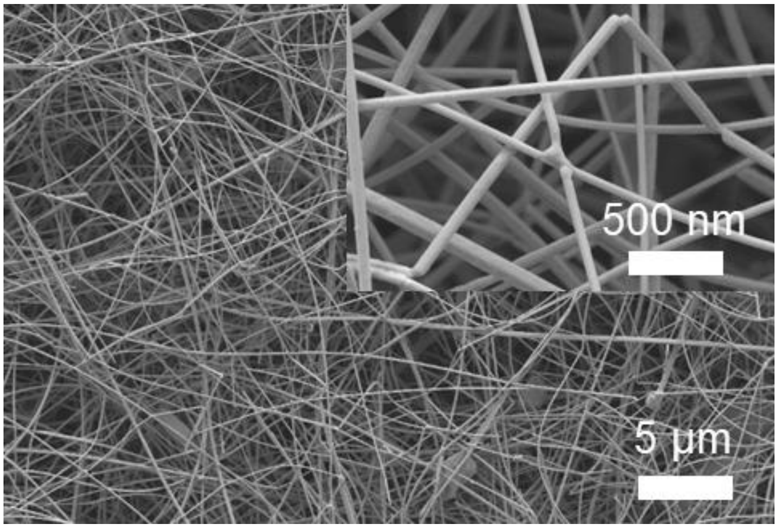

Fig. S1. SEM image of Ag-aerogel with different magnification.

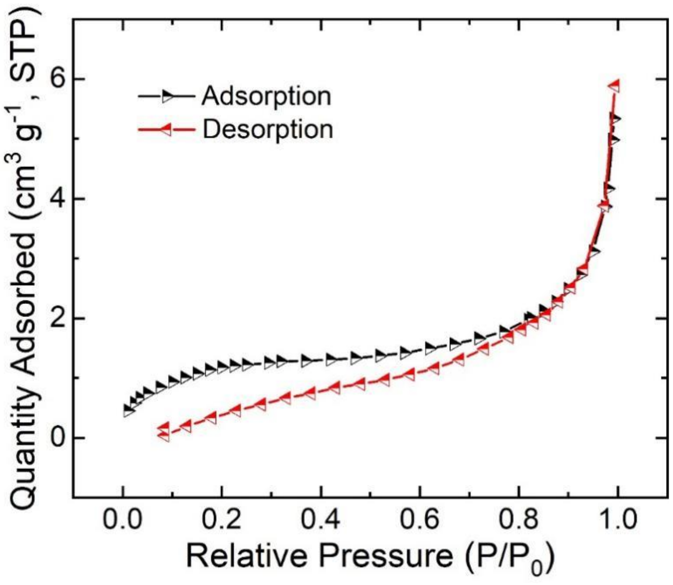

Fig. S2. N_2 adsorption/desorption isotherms for Ag aerogel at 77 K. The specific surface area is 4.7 m^2 g^{-1} calculated by Brunauer-Emmett-Teller (BET) method.

Table S1. The specific surface area of Ag_2Se and Ag_2Se@PI aerogel based on Brunauer-Emmett-Teller (BET) and mercury intrusion porosimetry (MIP) methods.

Sample	Measured	Specific surface area (m^2/g)
Ag_2Se aerogel	BET	4.4
Ag_2Se aerogel	MIP	1.4
Ag_2Se@PI aerogel	BET	4.1
Ag_2Se@PI aerogel	MIP	1.2

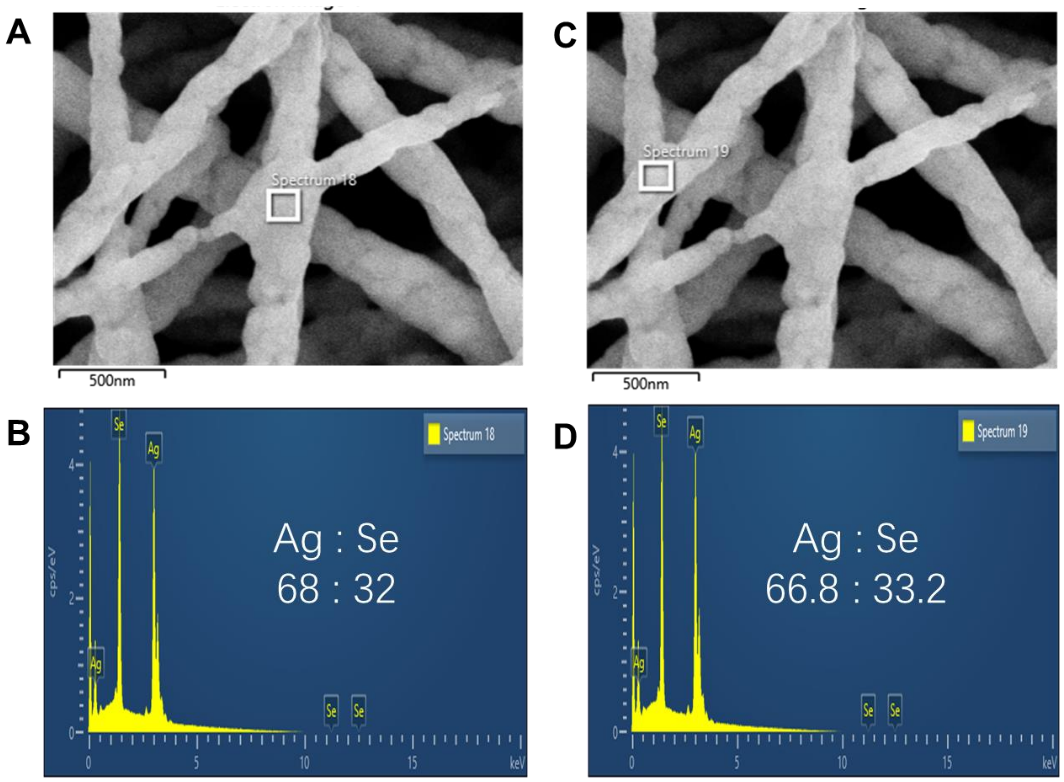

Fig. S3. SEM-EDS images of Ag_2Se-aerogel.

The principle of the Archimedes method is given as follows: The weights of the Ag_2Se aerogel sample in air ($m_{\text{aerogel-air}}$), Ag_2Se aerogel sample in water ($m_{\text{aerogel-water}}$). Then the Ag_2Se aerogel's volume V_{aerogel} can be calculated using

$$V_{aerogel} = \frac{m_{aerogel-air} - m_{aerogel-water}}{\rho_{water}}$$

where ρ_{water} is the density of water. The Ag_2Se aerogel's ρ_{aerogel} can be calculated using

$$\rho_{aerogel} = \frac{m_{aerogel-air}}{V_{aerogel}}$$

So, the porosity of the Ag_2Se aerogel can be expressed as

$$Porosity = \frac{\rho_{aerogel}}{\rho_{crystal}} \times 100\%$$

where ρ_{crystal} is the calculated density of Ag_2Se single crystals.

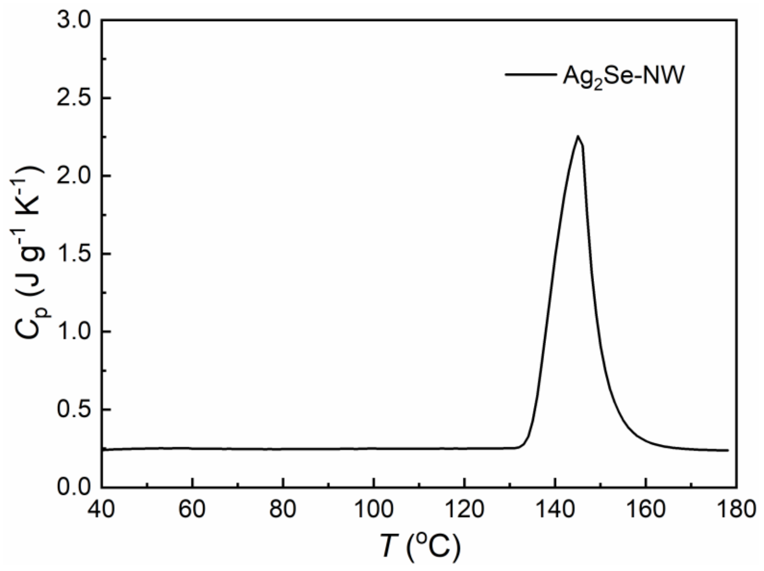

Fig. S4. Temperature dependence of heat capacity for Ag_2Se-NW.

Table S2. Comparison of the thermoelectric parameters of aerogel thermoelectric materials.

Materials	Method	Density (g/cm^3)	σ (S/cm)	S (μV/K)	κ (W/mK)	zT	Refs.
PEDOT:PSS	Supercritical CO_2 drying	0.0336	2.35	22	0.042	2×10^{-3}	(*32*)
PEDOT:PSS	Freezing drying	0.3×10^{-3}	70	16	/	/	(*18*)
PEDOT:PSS/CNT	Freezing drying	0.061	2.5~4.4	51-52	0.07~0.14	0.02	(*21*)
PEDOT:PSS/SWCNT	Freezing drying	/	3.7	38.9	0.074	0.028	(*35*)
PEDOT:PSS/CNT/Ag	Freezing drying	/	6.7	58	0.093	7.6×10^{-3}	(*20*)
BC/PEDOT/SWCNT	Directional freezing		0.8	28.6	0.0322	0.012	(*36*)
PEDOT:PSS/Te-NW	Freezing drying + vapor post-treatment	/	105	32	0.1	0.02	(*26*)
PEDOT:PSS/Bi_2Te_3-NW	Hydrothermal + hot drying	/	100	24.2	0.047	0.048	(*52*)
PEDOT:PSS/GOPS/NFC	Freezing drying	0.0108		20	/	/	(*63*)
P3HT-PS	Freezing drying	0.9×10^{-3}	10^{-5}	10^4	0.028	7×10^{-3}	(*19*)
CNT	Solvent evaporation	0.035	4.02	32.6	0.17-0.42	7.6×10^{-4}	(*64*)
CNT	Chemical vapor deposition	0.011	1.23	18.2	0.02	6×10^{-4}	(*65*)
rGO/CNT	Freezing drying	0.012	6.7	29	0.021	8×10^{-3}	(*13*)
CNT/Ag	Freezing drying	0.13	10.61	54	0.06	0.011	(*33*)
MWCNT/RF	Freezing drying	0.117	3.7	47	0.075	2.4×10^{-3}	(*34*)
MWCNT/GO	Freezing drying	0.024	0.57	70	0.056	0.001	(*66*)
GO	Freezing drying	0.007	0.06-0.3	29.5	0.021	0.048	(*37*)
GO	Directional freeze-carbonization	/	29	20	0.294	0.036	(*67*)
rGOTEA	Chemical agent-assisted bottom-up	5.4×10^{-3}	0.96	6.9~19.5	0.028	6.7×10^{-5}	(*68*)
$Bi_{0.5}Sb_{1.5}Te_3$ aerogel	Supercritical CO_2 drying	0.06	100	-40	0.12	0.058	(*29*)
Ag_2Se foam	Two-step impregnation	0.28	8.6	-129	0.04	0.11	(*30*)
Ag_2Se nanowire	Hydrothermal + Supercritical CO_2 drying	0.04~0.54	26.2	-117	0.61	0.17	This work

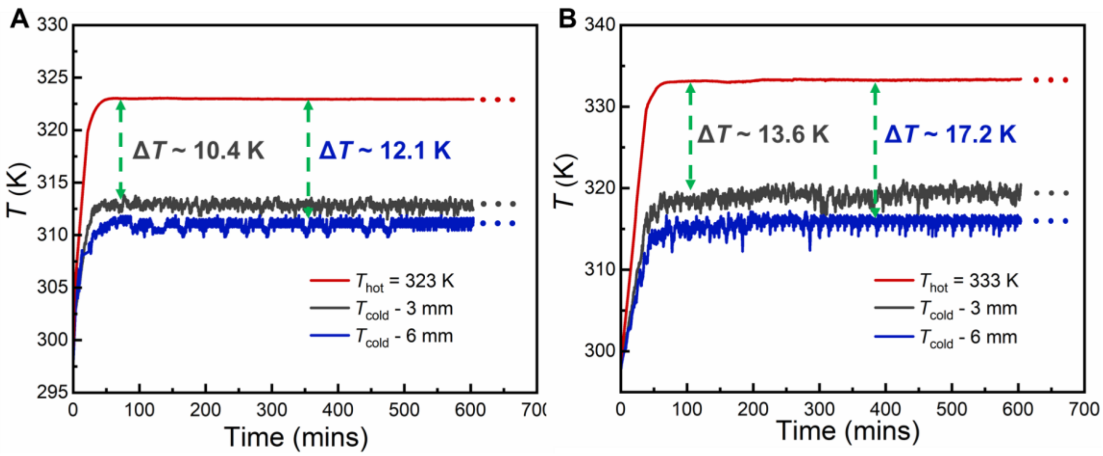

Fig. S5. Time-dependent temperature curves of the Ag_2Se-NW aerogel. Time-dependent temperature curves under air exposure showing the ability of the Ag_2Se aerogel to establish a temperature difference (ΔT) at the hot-end temperature fixed at (a) 323 K and (b) 333 K. The T_{hot}, T_{cold}-3 mm and T_{cold}-6 mm represent the hot side temperature, the cold side temperature of Ag_2Se aerogel with the size of 15 mm × 3 mm × 1 mm and 15 mm × 6 mm × 1 mm in which 3 mm and 6 mm are set as the direction of heat flow, respectively.

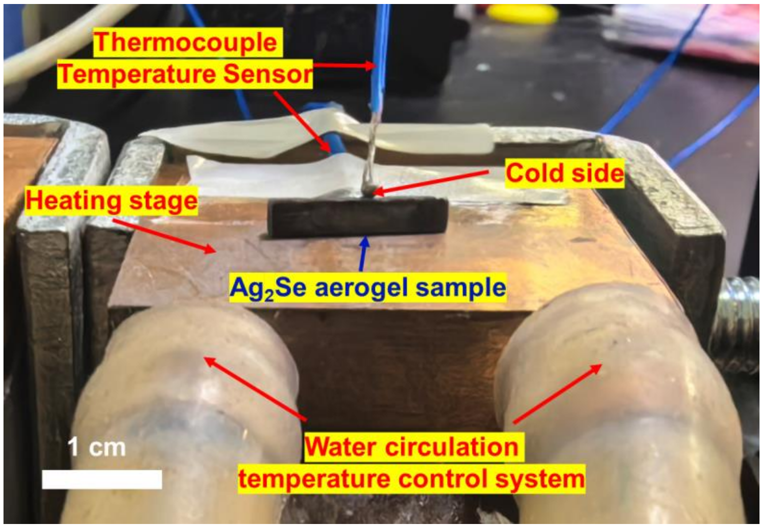

Fig. S6. The photo of the experimental setup for temperature difference measurement.

Table S3. Steady-state temperature-gradient performance of Ag_2Se aerogel legs.

Stage T (K)	Height (mm)	$\Delta T_{measured}$ (C)	ΔT_{theory} (K)	$\varphi_{thermal}$ (%)
323	3	10.7	25	42.8
323	6	12.1	25	48.4
333	3	13.6	35	38.9
333	6	17.2	35	49.1

Table S4. The simulation conditions and parameters

Parameters	values	Source
Thermal conductivity of Ag_2Se aerogel	0.04 W m^{-1} K^{-1}	Measurement
Thermal conductivity of copper	400 W m^{-1} K^{-1}	COMSOL
Resistivity of Ag_2Se aerogel	0.38×10^{-3} Ω m	Measurement
Resistivity of copper	1.7×10^{-8} Ω m	COMSOL
Seebeck coefficient of Ag_2Se network	-122 μV K^{-1}	Measurement
Heat transfer coefficient of air	6 W m^{-2} K^{-1}	COMSOL
Medial natural convection pressure (air)	1 atm	COMSOL

Table S5. The output voltage depends on the hot-side temperature for three kinds of cupper electrode.

Electrode width (mm)	Hot-side temperature (K)	Output voltage (mV)
0.1	303	0.79
	313	1.76
	323	2.78
	333	3.86
	343	4.98
	353	6.12
1	303	0.60
	313	1.49
	323	2.43
	333	3.41
	343	4.43
	353	5.47
15	303	0.49
	313	1.39
	323	2.33
	333	3.30
	343	4.31
	353	5.33

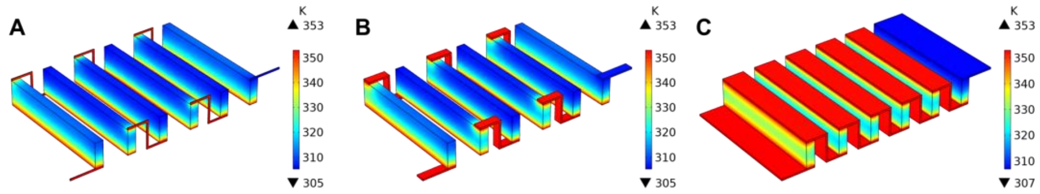

Fig. S7. Computational simulation temperature distribution. Temperature distribution of the Ag_2Se aerogel-based generator with different kinds of electrode widths, and the dimension of the Ag_2Se aerogel legs is about $3\times3\times10$ mm^3.

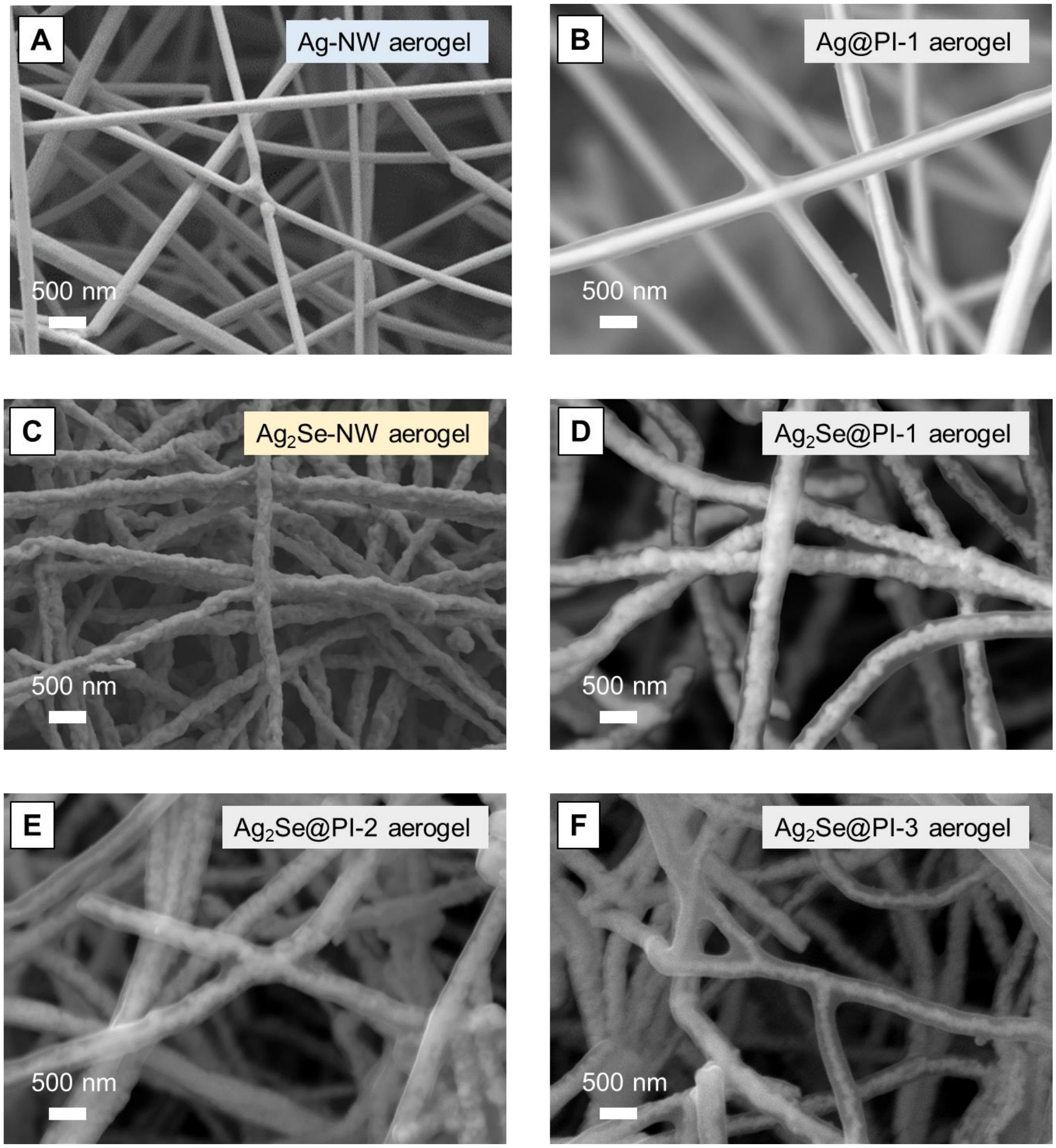

Fig. S8. Microscopy study of aerogel structure. The SEM images of (a) Ag-NW aerogel, (b) Ag@PI-1 aerogel, (c) Ag_2Se-NW aerogel, (d) ~ (f) Ag_2Se aerogel encapsulated with different content of PI, named Ag_2Se@PI-1 aerogel, Ag_2Se@PI-2 aerogel, and Ag_2Se@PI-3 aerogel.

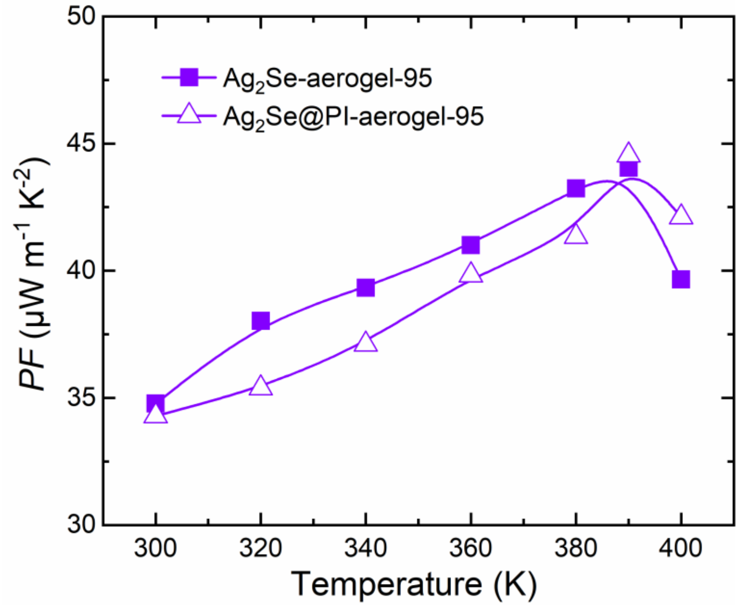

Fig. S9. Thermoelectric properties testing. Temperature-dependent power factor (*PF*) of the Ag_2Se-aerogels-95 and Ag_2Se@PI-aerogels-95.

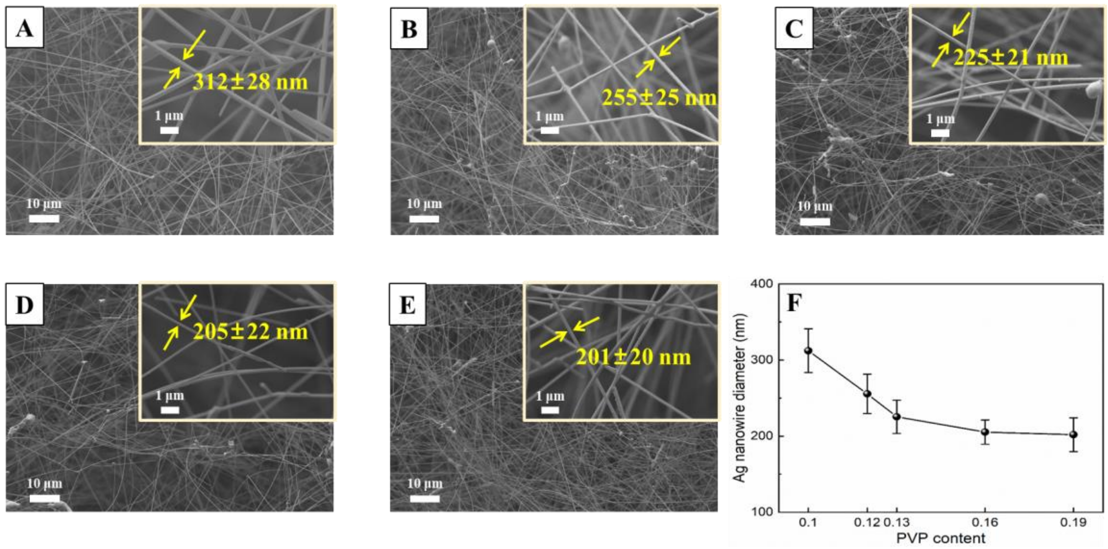

Fig. S10. Diameter-depended microscopy study of Ag-NW aerogels. (A) ~ (E) SEM images of Ag-NW aerogels synthesized with varying PVP content, with magnified insets highlighting morphological differences. (F) The corresponding plot shows the variation in Ag-NW diameter as a function of PVP content.

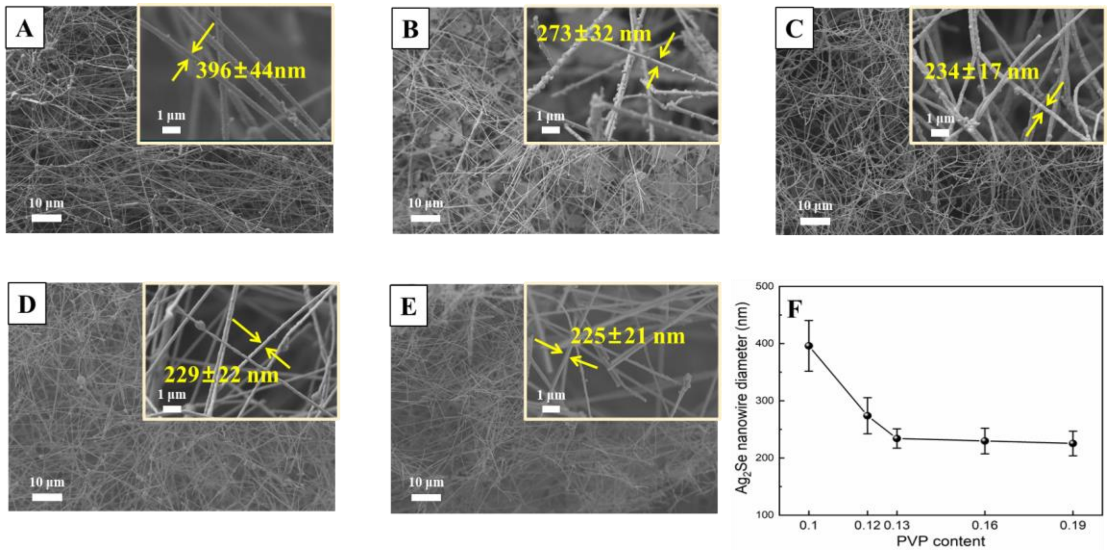

Fig. S11. Diameter-depended microscopy study of Ag_2Se-NW aerogels. (A) ~ (E) SEM images of Ag_2Se-NW aerogels prepared with varying PVP content, with magnified insets illustrating the morphological differences. (F) The corresponding plot depicts the variation in Ag_2Se-NW diameter as a function of PVP content.

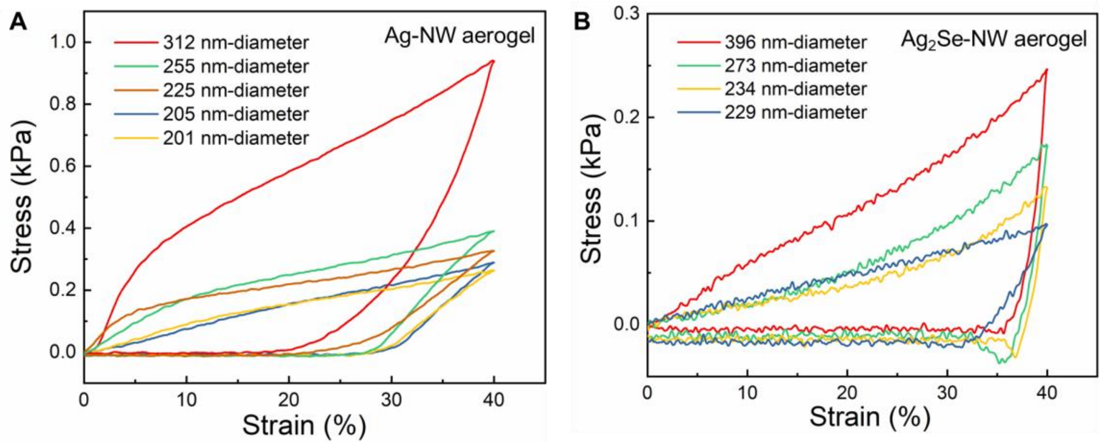

Fig. S12. Mechanical compression testing. Stress–strain curves of (A) Ag-NW aerogels and (B) Ag_2Se-NW aerogels with different diameters under compressive strain up to 40%.

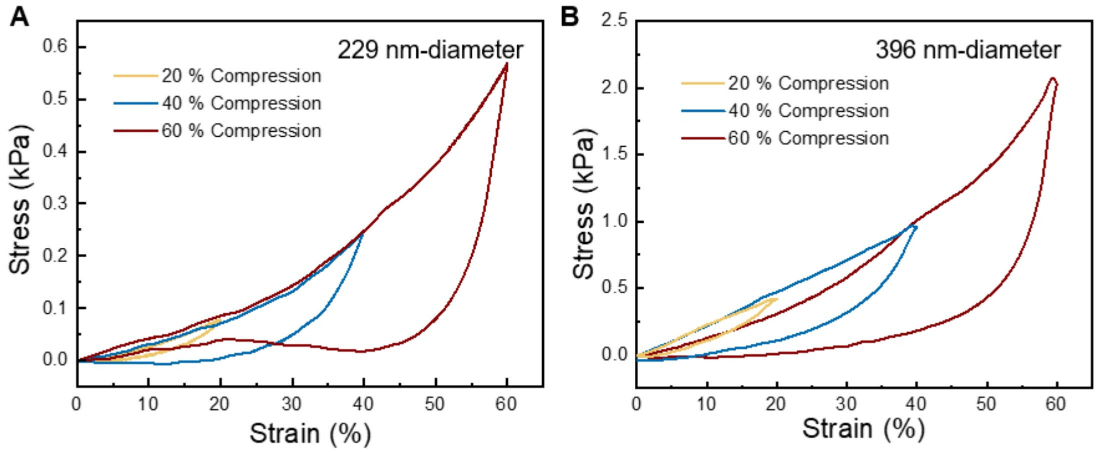

Fig. S13. Stress-strain curves testing. The Stress-strain curves of (A) 225 nm-diameter Ag_2Se@PI aerogel and (B) 396 nm-diameter Ag_2Se@PI aerogel at special strains (20%, 40%, and 60% compression).

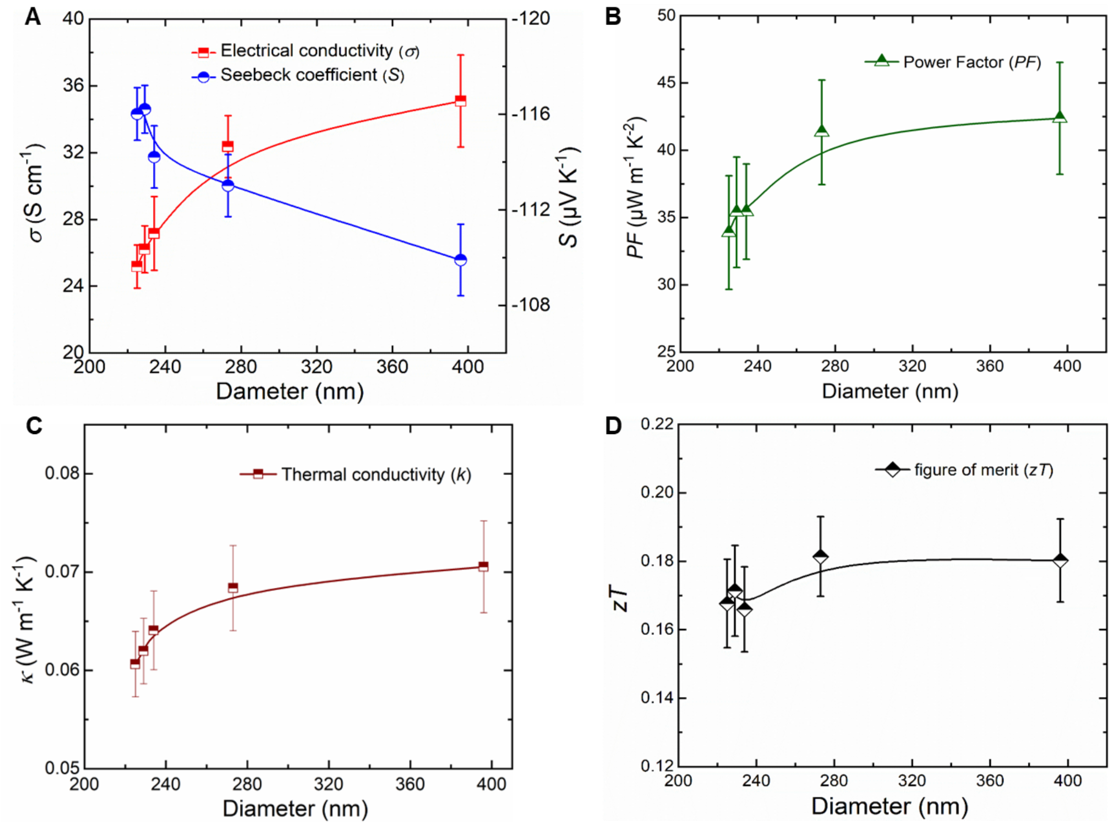

Fig. S14. Diameter-depended thermoelectric properties. (A) Electrical conductivity (σ) and Seebeeck coefficient (S), (B) power factor (PF), (C) thermal conductivity (κ), and (D) figure-of-merit zT of Ag_2Se aerogels as a function of Ag_2Se-NW diameter.

Movie S1. Stress-strain cycle testing of Ag_2Se@PI aerogel. The video shows the cycle compression tests under 40% strain of Ag_2Se@PI aerogel.

REFERENCES

1. S. Hong, Y. Gu, J. K. Seo, J. Wang, P. Liu, Y. S. Meng, S. Xu, R. Chen, Wearable thermoelectrics for personalized thermoregulation. *Sci. Adv.* **5**, eaaw0536 (2019).

2. Y. Wang, C. Zhu, R. Pfattner, H. Yan, L. Jin, S. Chen, F. Molina-Lopez, F. Lissel, J. Liu, N. I. Rabiah, Z. Chen, J. W. Chung, C. Linder, M. F. Toney, B. Murmann, Z. Bao, A highly stretchable, transparent, and conductive polymer. *Sci. Adv.* **3**, e1602076 (2017).

3. M. Wu, Z. Shao, N. Zhao, R. Zhang, G. Yuan, L. Tian, Z. Zhang, W. Gao, H. Bai, Biomimetic, knittable aerogel fiber for thermal insulation textile. *Science* **382**, 1379–1383 (2023).

4. J. Guo, S. Fu, Y. Deng, X. Xu, S. Laima, D. Liu, P. Zhang, J. Zhou, H. Zhao, H. Yu, S. Dang, J. Zhang, Y. Zhao, H. Li, X. Duan, Hypocrystalline ceramic aerogels for thermal insulation at extreme conditions. *Nature* **606**, 909–916 (2022).

5. X. Lu, M C Arduini-Schuster, J. Kuhn, O. Nilsson, J. Fricke, R W Pekala, Thermal conductivity of monolithic organic aerogels. *Science* **255**, 971–972 (1992).

6. L. Liang, X. Wang, Z. Liu, G. Sun, G. Chen, Recent advances in organic, inorganic, and hybrid thermoelectric aerogels. *Chin. Phys. B* **31**, 027903 (2022).

7. Y. Zhang, S. Park, Flexible organic thermoelectric materials and devices for wearable green energy harvesting. *Polymers* **11**, 909 (2019).

8. C. I. Idumah, Novel trends in polymer aerogel nanocomposites. *Polym. Plast. Technol. Mater.*, **60**, 1519–1531 (2021).

9. B. Liang, B. Huang, J. He, R. Yang, C. Zhao, B.-R. Yang, A. Cao, Z. Tang, X. Gui, Direct stamping multifunctional tactile sensor for pressure and temperature sensing. *Nano Res.* **15**, 3614–3620 (2021).

10. B. Liang, W. Chen, Z. He, R. Yang, Z. Lin, H. Du, Y. Shang, A. Cao, Z. Tang, X. Gui, Highly sensitive, flexible MEMS based pressure sensor with photoresist insulation layer. *Small* **13**, 1702422 (2017).

11. L. Su, H. Wang, M. Niu, S. Dai, Z. Cai, B. Yang, H. Huyan, X. Pan, Anisotropic and hierarchical SiC@SiO_2 nanowire aerogel with exceptional stiffness and stability for thermal superinsulation. *Science* **6**, eaay6689 (2020).

12. L. Su, S. Jia, J. Ren, X. Lu, S.-W. Guo, P. Guo, Z. Cai, D. Lu, M. Niu, L. Zhuang, K. Peng, H. Wang, Strong yet flexible ceramic aerogel. *Nat. Commun.* **14**, 7057 (2023).

13. D. Tan, J. Zhao, C. Gao, H. Wang, G. Chen, D. Shi, Carbon nanoparticle hybrid aerogels: 3D double-interconnected network porous microstructure, thermoelectric, and solvent-removal functions. *ACS Appl. Mater. Interfaces* **9**, 21820–21828 (2017).

14. Z. H. Liu, Y. D. Ding, F. Wang, Z. P. Deng, Thermal insulation material based on SiO_2 aerogel. *Construct. Build Mater.* **122**, 548–555 (2016).

15. F. Guo, Y. Jiang, Z. Xu, Y. Xiao, B. Fang, Y. Liu, W. Gao, P. Zhao, H. Wang, C. Gao, Highly stretchable carbon aerogels. *Nat. Commun.* **9**, 881 (2018).

16. Y. Ma, Y. Yue, H. Zhang, F. Cheng, W. Zhao, J. Rao, S. Luo, J. Wang, X. Jiang, Z. Liu, N. Liu, Y. Gao, 3D synergistical MXene/reduced graphene oxide aerogel for a piezoresistive sensor. *ACS Nano* **12**, 3209–3216 (2018).

17. Y. Y. Jiang, Y. J. Zhu, F. Chen, J. Wu, Solvothermal synthesis of submillimeter ultralong hydroxyapatite nanowires using a calcium oleate precursor in a series of monohydroxy alcohols. *Construct. Build Mater.* **41**, 6098–6102 (2015).

18. M. P. Gordon, E. W. Zaia, P. Zhou, B. Russ, N. E. Coates, A. Sahu, J. J. Urban, Soft PEDOT:PSS aerogel architectures for thermoelectric applications. *J. Appl. Polym. Sci.* **134**, 10.1002/app.44070 (2016).

19. N. Okada, K. Sato, M. Yokoo, E. Kodama, S. Kanehashi, T. Shimomura, Thermoelectric properties of poly(3-hexylthiophene) nanofiber aerogels with a giant Seebeck coefficient. *ACS Appl. Polym. Mater.* **3**, 455–463 (2020).

20. X. Sun, Y. Wei, J. Li, J. Zhao, L. Zhao, Q. Li, Ultralight conducting PEDOT:PSS/carbon nanotube aerogels doped with silver for thermoelectric materials. *Sci. China Mater.* **60**, 159–166 (2017).

21. X. Wang, L. Liang, H. Lv, Y. Zhang, G. Chen, Elastic aerogel thermoelectric generator with vertical temperature-difference architecture and compression-induced power enhancement. *Nano Energy* **90**, 106577 (2021).

22. S. Gupta, R. Meek, Highly efficient thermo-electrochemical energy harvesting from graphene–carbon nanotube 'hybrid' aerogels. *Appl. Phys. A* **126**, 704 (2020).

23. B. Feng, Study on the optimization of thermoelectric properties of BiCuSeO ceramics by highly insulating/adiabatic SiO_2 aerogel dispersion. *J. Mater. Sci. Mater. Electron.* **32**, 25473–25480 (2021).

24. R. Du, Y. Hu, R. Hübner, J.-O. Joswig, X. Fan, K. Schneider, A. Eychmüller, Specific ion effects directed noble metal aerogels: Versatile manipulation for electrocatalysis and beyond. *Sci. Adv.* **5**, eaaw4590 (2019).

25. W. Zhu, P. Wang, Z. Chen, C. Xu, Y. Jiao, M. Li, Y. Huang, A one-pot self-assembled AgNW aerogel electrode with ultra-high electric conductivity for intrinsically 500% super-stretchable high-performance Zn–Ag batteries. *J. Mater. Chem. A* **10**, 10780–10789 (2022).

26. X. Wang, P. Liu, Q. Jiang, W. Zhou, J. Xu, J. Liu, Y. Jia, X. Duan, Y. Liu, Y. Du, F. Jiang, Efficient DMSO-vapor annealing for enhancing thermoelectric performance of PEDOT:PSS-based aerogel. *ACS Appl. Mater. Interfaces* **11**, 2408–2417 (2019).

27. X. Zhang, D. Chang, J. Liu, Y. Luo, Conducting polymer aerogels from supercritical CO_2 drying PEDOT-PSS hydrogels. *J. Mater. Chem.* **20**, 5080–5085 (2010).

28. Z. U. Khan, J. Edberg, M. M. Hamedi, R. Gabrielsson, H. Granberg, L. Wagberg, I. Engquist, M. Berggren, X. Crispin, Thermoelectric polymers and their elastic aerogels. *Adv. Mater.* **28**, 4556–4562 (2016).

29. S. Ganguly, C. Zhou, D. Morelli, J. Sakamoto, S. L. Brock, Synthesis and characterization of telluride aaerogels: Effect of gelation on thermoelectric performance of Bi_2Te_3 and $Bi_{2-x}Sb_xTe_3$ nanostructures. *J. Phys. Chem. C* **116**, 17431–17439 (2012).

30. Y. Liu, X. Wang, S. Hou, Z. Wu, J. Wang, J. Mao, Q. Zhang, Z. Liu, F. Cao, Scalable-produced 3D elastic thermoelectric network for body heat harvesting. *Nat. Commun.* **14**, 3058 (2023).

31. Y. Lei, R. Qi, M. Chen, H. Chen, C. Xing, F. Sui, L. Gu, W. He, Y. Zhang, T. Baba, T. Baba, H. Lin, T. Mori, K. Koumoto, Y. Lin, Z. Zheng, Microstructurally tailored thin β-Ag_2Se films toward commercial flexible thermoelectrics. *Adv. Mater.* **34**, e2104786 (2022).

32. E. D. Kolb, R. A. Laudise, The solubility of trigonal Se in Na_2S solutions and the hydrothermal growth of Se. *J. Cryst. Growth* **8**, 191–196 (1971).

33. Q. Gao, W. Wang, Y. Lu, K. Cai, Y. Li, Z. Wang, M. Wu, C. Huang, J. He, High power factor Ag/Ag_2Se composite films for flexible thermoelectric generators. *ACS Appl. Mater. Interfaces* **13**, 14327–14333 (2021).

34. X. Zhao, Z. Chen, H. Zhuo, Y. Hu, G. Shi, B. Wang, H. Lai, S. Araby, W. Han, X. Peng, L. Zhong, Thermoelectric generator based on anisotropic wood aerogel for low-grade heat energy harvesting. *J. Mater. Sci. Technol.* **120**, 150–158 (2022).

35. S. Hou, Y. Liu, L. Yin, C. Chen, Z. Wu, J. Wang, Y. Luo, W. Xue, X. Liu, Q. Zhang, F. Cao, High performance wearable thermoelectric generators using Ag_2Se films with large carrier mobility. *Nano Energy* **87**, 106223 (2021).

36. S. Hou, Y. Liu, Y. Luo, X. Wang, L. Yin, X. Sun, Z. Wu, J. Wang, M. Li, Z. Chen, Y. Wang, J. Sui, J. Mao, X. Liu, Q. Zhang, F. Cao, High-performance, thin-film thermoelectric generator with self-healing ability for body-heat harvesting. *Cell Rep. Phys. Sci.* **3**, 101146 (2022).

37. S. Huang, T. R. Wei, H. Chen, J. Xiao, M. Zhu, K. Zhao, X. Shi, Thermoelectric Ag_2Se: Imperfection, homogeneity, and reproducibility. *ACS Appl. Mater. Interfaces* **13**, 60192–60199 (2021).

38. M. S. Toivonen, A. Kaskela, O. J. Rojas, E. I. Kauppinen, O. Ikkala, Ambient-dried cellulose nanofibril aerogel membranes with high tensile strength and their use for aerosol collection and templates for transparent, flexible devices. *Adv. Funct. Mater.* **25**, 6618–6626 (2015).

39. J. Wan, J. Zhang, J. Yu, J. Zhang, Cellulose aerogel membranes with a tunable nanoporous network as a matrix of gel polymer electrolytes for safer lithium-ion batteries. *ACS Appl. Mater. Interfaces* **9**, 24591–24599 (2017).

40. L. Su, H. Wang, M. Niu, X. Fan, M. Ma, Z. Shi, S. W. Guo, Ultralight, recoverable, and high-temperature-resistant SiC nanowire aerogel. *ACS Nano* **12**, 3103–3111 (2018).

41. C. Yu, Y. S. Song, Analysis of thermoelectric energy harvesting with graphene aerogel-supported form-stable phase change materials. *Nanomaterials* **11**, 2192 (2021).

42. E.-Y. Choi, C. A. Wray, C. Hu, W. Choe, Highly tunable metal–organic frameworks with open metal centers. *CrstEngComm* **330**, 581–583 (2000).

43. C. Jiang, Y. Ding, K. Cai, L. Tong, Y. Lu, W. Zhao, P. Wei, Ultrahigh performance of n-type Ag_2Se films for flexible thermoelectric power generators. *ACS Appl. Mater. Interfaces* **12**, 9646–9655 (2020).

44. Y. Ding, Y. Qiu, K. Cai, Q. Yao, S. Chen, L. Chen, J. He, High performance n-type Ag_2Se film on nylon membrane for flexible thermoelectric power generator. *Nat. Commun.* **10**, 841 (2019).

45. S. Lin, L. Guo, X. Wang, Y. Liu, Y. Wu, R. Li, H. Shao, M. Jin, Revealing the promising near-room-temperature thermoelectric performance in Ag_2Se single crystals. *J. Mater.* **9**, 754–761 (2023).

46. W. Thongkham, C. Lertsatitthanakorn, K. Jiramitmongkon, K. Tantisantisom, T. Boonkoom, M. Jitpukdee, K. Sinthiptharakoon, A. Klamchuen, M. Liangruksa, P. Khanchaitit, Self-assembled three-dimensional Bi_2Te_3 nanowire-PEDOT:PSS hybrid nanofilm network for ubiquitous thermoelectrics. *ACS Appl. Mater. Interfaces* **11**, 6624–6633 (2019).

47. C. Meng, C. Liu, S. Fan, A promising approach to enhanced thermoelectric properties using carbon nanotube networks. *Adv. Mater.* **22**, 535–539 (2010).

48. Q. Zhou, K. Zhu, J. Li, Q. Li, B. Deng, P. Zhang, Q. Wang, C. Guo, W. Wang, W. Liu, Leaf-inspired flexible tthermoelectric generators with high temperature difference utilization ratio and output power in ambient air. *Adv. Sci.* **8**, 2004947 (2021).

49. F. Suarez, A. Nozariasbmarz, D. Vashaee, M. C. Öztürk, Designing thermoelectric generators for self-powered wearable electronics. *Energy Environ. Sci.* **9**, 2099–2113 (2016).

50. Y. Liu, S. Hou, X. Wang, L. Yin, Z. Wu, X. Wang, J. Mao, J. Sui, X. Liu, Q. Zhang, Z. Liu, F. Cao, Passive radiative cooling enables improved performance in wearable thermoelectric generators. *Small* **18**, e2106875 (2022).

51. N. Yanagishima, S. Kanehashi, H. Saito, K. Ogino, T. Shimomura, Thermoelectric properties of PEDOT:PSS aerogel secondary-doped in supercritical CO_2 atmosphere with low thermal conductivity. *Polymer* **206**, 122912 (2020).

52. X. Qi, T. Miao, C. Chi, G. Zhang, C. Zhang, Y. Du, M. An, W.-G. Ma, X. Zhang, Ultralight PEDOT:PSS/graphene oxide composite aerogel sponges for electric power harvesting from thermal fluctuations and moist environment. *Nano Energy* **77**, 105096 (2020).

53. M. H. Tsai, I. H. Tseng, J. C. Chiang, J. J. Li, Flexible polyimide films hybrid with functionalized boron nitride and graphene oxide simultaneously to improve thermal conduction and dimensional stability. *ACS Appl. Mater. Interfaces* **6**, 8639–8645 (2014).

54. G. Chen, R. Rastak, Y. Wang, H. Yan, V. Feig, Y. Liu, Y. Jiang, S. Chen, F. Lian, F. Molina-Lopez, L. Jin, K. Cui, J. W. Chung, E. Pop, C. Linder, Z. Bao, Strain- and Strain-rate-invariant conductance in a stretchable and compressible 3D conducting polymer foam. *Matter* **1**, 205–218 (2019).

55. W. Zheng, X. Wang, B. Liu, C. Yang, A. Zhang, A multi-functional foam with an integrated antique square pedestal structure exhibiting broadband and stable microwave absorption performance. *Adv. Mater. Technol.* **10**, 2400147 (2025).

56. X. Zhao, W. Wang, Z. Wang, J. Wang, T. Huang, J. Dong, Q. Zhang, Flexible PEDOT:PSS/polyimide aerogels with linearly responsive and stable properties for piezoresistive sensor applications. *Chem. Eng. J.* **395**, 125115 (2020).

57. F. Peng, Y. Fang, Z. Han, W. Zhu, M. Li, "Lightweight silver nanowire aerogel for electromagnetic interference shielding," in *22nd International Conference on Electronic Packaging Technology* (ICEPT, 2021), pp. 1–4.

58. X. Sun, J. Zhao, L. Zhao, J. Wu, Q. Li, Thermoelectric performance of conducting aerogels based on carbon nanotube/silver nanocomposites with ultralow thermal conductivity. *RSC Adv.* **6**, 109878–109884 (2016).

59. L. Zhao, J. Zhao, X. Sun, Q. Li, J. Wu, A. Zhang, Enhanced thermoelectric properties of hybridized conducting aerogels based on carbon nanotubes and pyrolyzed resorcinol–formaldehyde resin. *Synth. Met.* **205**, 64–69 (2015).

60. H. Li, Z. Ding, Q. Zhou, J. Chen, Z. Liu, C. Du, L. Liang, G. Chen, Harness hhigh-temperature thermal energy via elastic thermoelectric aerogels. *Nanomicro Lett.* **16**, 151 (2024).

61. L. Chen, J. Lou, Y. Zong, Z. Liu, Y. Jiang, W. Han, Wood-like aerogel for thermoelectric generators based on BC/PEDOT/SWCNT. *Cellul.* **30**, 3141–3152 (2023).

62. Y. Yang, W. Yan, A. Anand, D. Fuan, E. Nguyen, C. Felder, M. Kulkarni, J. Qiu, 3D N-doped crumpled graphene aerogels for thermoelectric energy harvesting and highly sensitive piezoresistive sensing. *Carbon* **232**, 119827 (2025).

63. S. Han, F. Jiao, Z. U. Khan, J. Edberg, S. Fabiano, X. Crispin, Thermoelectric polymer aerogels for pressure-temperature sensing applications. *Adv. Funct. Mater.* **27**, 1703549 (2017).

64. M. H. Lee, Y. H. Kang, J. Kim, Y. K. Lee, S. Y. Cho, Freely shapable and 3D porous carbon nanotube foam using rapid solvent evaporation method for flexible thermoelectric power generators. *Adv. Energy Mater.* **9**, 1900914 (2019).

65. J. Chen, X. Gui, Z. Wang, Z. Li, R. Xiang, K. Wang, D. Wu, X. Xia, Y. Zhou, Q. Wang, Z. Tang, L. Chen, Superlow thermal conductivity 3D carbon nanotube network for thermoelectric applications. *ACS Appl. Mater. Interfaces* **4**, 81–86 (2012).

66. L. Zhao, X. Sun, Z. Lei, J. Zhao, J. Wu, Q. Li, A. Zhang, Thermoelectric behavior of aerogels based on graphene and multi-walled carbon nanotube nanocomposites. *Compos. Part B Eng.* **83**, 317–322 (2015).

67. Y. Nie, Y. Hu, N. Xin, Y. Li, X. Zhao, M. Zhang, G. Tang, Cost-effective graphite aerogel for high-temperature thermoelectrics: Synergizing ultra-high electrical conductivity and thermal insulation. *J. Colloid Interface Sci.* **697**, 137926 (2025).

68. P. He, J. Liu, Y. Wen, N. Shao, Q. Zhang, Highly robust 3D rGO aerogel with tunable thermoelectric effect for multifunctional sensing applications. *ACS Appl. Mater. Interfaces* **17**, 24339–24350 (2025).
